# Role of CD25 on resting Treg immune cell in mediating the effect of stearate biosynthesis microbiome pathway on lung adenocarcinoma

**DOI:** 10.1097/MD.0000000000047505

**Published:** 2026-01-30

**Authors:** Lishi Yang, Lei Li, Bin Liang, Jihua Yu, Jianxiong Wang, Fuhua Sun

**Affiliations:** a Department of Oncology, The Affiliated Hospital of Southwest Medical University, Luzhou, Sichuan, P. R. China; b Department of Rehabilitation, The Affiliated Hospital of Southwest Medical University, Luzhou, Sichuan, P. R. China; c Department of Rehabilitation Medicine, Southwest Medical University, Luzhou, Sichuan, P. R. China; d Rehabilitation Medicine and Engineering Key Laboratory of Luzhou, Luzhou, Sichuan, P. R. China.

**Keywords:** gut microbiota, immune cell, lung adenocarcinoma

## Abstract

Gut microbiota (GM) often influences the development of diseases by regulating the immune system. The causal relationship between GM and lung adenocarcinoma (LUAD) and whether it can be mediated by immune cells remains unclear. We conducted two-step, two-sample Mendelian randomization (MR) analyses. The data of GM, LUAD and immune cells for analysis were derived from publicly available genetic data. The causal relationship between GM and LUAD, as well as the mediation effect of immune cells in the association between GM and LUAD were estimated using inverse variance weighted, MR-Egger and weighted median. Sensitivity analyses were implemented with Cochran *Q* test, MR-Egger intercept test, MR-PRESSO. MR analyses determined the protective effects of PWY-5989 (stearate biosynthesis II bacteria and plants) on LUAD. In addition, CD25 on resting regulatory T cell (Treg) was negatively correlated with LUAD. Of note, the mediation MR illustrated that in the presence of CD25 on resting Treg, PWY-5989 can promote the risk of LUAD by inhibiting the expression of CD25 on resting Treg. The study suggested a causal relationship between PWY-5989 and LUAD, which may be mediated by CD25 on resting Treg.

## 1. Introduction

Lung cancer is one of the most common malignancies and the leading cause of tumor-related deaths worldwide.^[1]^ There are 2 major categories of lung cancer: Non-small cell lung cancer (NSCLC) and small cell lung cancer. Among them, NSCLC is the main pathological type of lung cancer, accounts for 85%.^[1]^ In NSCLC, adenocarcinoma is the most predominant type.^[2]^ The treatment of lung adenocarcinoma (LUAD) has made breakthroughs, such as surgery, radiotherapy, chemotherapy, targeted therapy, and immunotherapy, however, the overall prognosis of LUAD patients is still poor.^[3]^ There is an urgent need for reliable and easily detectable potentially modifiable risk factors to early diagnosis and treatment of LUAD.

There are diverse and extremely complex microbial communities in the gastrointestinal tract, called gut microbiota (GM).^[4]^ The literature has shown that the GM plays roles in the progression of tumors, including lung cancer.^[5]^ Several studies have explored the relationship between GM and lung cancer.^[6]^ For example, elevated levels of *Enterococcus*, *Veillonella*, *Fusobacterium* and *Bacteroides* have been linked to lung cancer.^[7,8]^ Zheng et al reported that high levels of *Bacillus* and *Akkermansia muciniphila* promote development of lung cancer.^[9]^ While, lower levels of *Escherichia*–*Shigella*, *Faecalibacterium*, *Dialister*, *Kluyvera* and *Enterobacter* are reported in lung cancer patients.^[8]^ The GM can affect the development, homeostasis and function of the immune system.^[10,11]^ The immune system is a key factor in the tumorigenesis and development process. Therefore, the interaction between the GM and the immune system is considered to play an important role in cancer immune surveillance.^[12]^ Recent studies further elucidate how specific microbial metabolites and pathways modulate T-cell differentiation and function within the tumor microenvironment.^[13–15]^

Mendelian randomization (MR) is a widely used analytical approach to explore the causal relationship between exposure and outcomes.^[16]^ MR analysis is an important alternative tool in the absence of randomized controlled studies, as it can provide reliable evidence for causality between exposure and disease risks.^[17–20]^ Studies have shown the partial role of GM in LUAD and the effect of GM on the immune system. However, whether GM can influence the progression of LUAD by modulating the immune system is unclear. Our research aimed to investigate the causality of GM on LUAD and whether it can be mediated by immune cells.

## 2. Methods and materials

### 2.1. Study design

**Figure 1.**
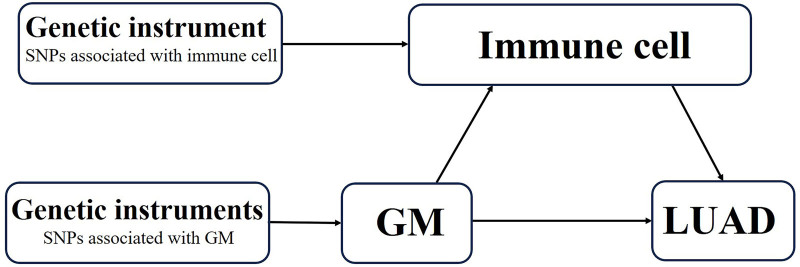
Diagrams depicting the connections investigated in the research. A two-step Mendelian randomization study of GM on LUAD mediated by immune cells. GM = gut microbiota, LUAD = lung adenocarcinoma, SNPs = single nucleotide polymorphisms.

To determine the relationship between GM and LUAD risk and whether immune cells may mediate this connection, we employed a two-step, two-sample MR analysis in the study. Figure 1 presents an overview of the study design. The study required no further ethical approval because it was based on publicly available data.

### 2.2. Data sources

The NHGRI-EBI GWAS Catalog (https://www.ebi.ac.uk/gwas/) provided summary statistics data for GM at the genus level, covering 412 (GCST90027446-GCST90027857) genera.^[21]^ The GWAS Catalog (https://gwas.mrcieu.ac.uk/) provided data on 731 immune cell characteristics.^[22]^ The GWAS statistics for LUAD in primary analysis were obtained from the TRICL https://www.ebi.ac.uk/gwas, comprising 3442 LUAD cases and 14,894 control subjects. Every participant in the research was European.

### 2.3. Selection of genetic instruments

A series of steps were performed to select eligible the NHGRI-EBI GWAS genetic variants. The filter condition of all single nucleotide polymorphisms (SNPs) as IVs was set to *P* < 1 × 10^−5^ in line with earlier studies.^[23]^ These SNPs were then clumped for independence using a linkage disequilibrium threshold of *r*^2^ < 0.001 within a 10,000 kb window, based on the European ancestry reference panel from the 1000 Genomes Project. To ensure the strength of the selected instruments and mitigate weak instrument bias, the F-statistic for each SNP was calculated using the formula *F* = *R*^2^(N − 2)/(1 − *R*^2^), where *R*^2^ represents the proportion of variance in the exposure explained by the SNP, and N is the sample size of the exposure GWAS. Only instruments with an F-statistic > 10 were retained for subsequent analyses.

### 2.4. MR analyses

All statistical analyses were performed using the “TwoSampleMR” package (Version 0.5.9) in the R program (Version 4.3.2; RStudio, Redmond). The random-effect inverse variance weighted (IVW) is the primarily used method in MR studies.^[24,25]^ Several other MR models, including weighted median^[26]^ and MR-Egger,^[27]^ were used as complementary methods to validate the robustness of the MR results. *P* < .05 indicated a substantial association between exposure and outcome. The Cochran *Q* test was employed to check whether heterogeneity existed. Cochran-*Q* derived *P < *.05 was recognized as existing heterogeneity.^[28]^ MR-PRESSO global test was used to select SNPs causing bias, and the MR-Egger intercept was implemented to determine horizontal pleiotropy.^[29,30]^ The MR-PRESSO outlier test was used to assess and correct horizontal pleiotropy.^[31]^ If significant outliers were detected by MR-PRESSO, they were removed, and the analysis was repeated. To find the high influence points influencing the pooled IVW estimates and further evaluate the reliability of the results, leave-one-out analysis was used. Bayesian weighted Mendelian randomization (BWMR) is a novel algorithm in MR analysis, which has good statistical efficiency and computational stability.^[32]^ In this study, we used BWMR to verify the robustness of the MR results.

## 3. Results

### 3.1. Effect of GM on LUAD

**Figure 2.**
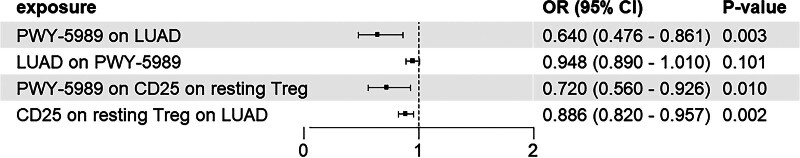
Forest plot to visualize the causal effects of CD25 on resting Treg with PWY-5989 and LUAD by IVW. CI = confidence interval, IVW = inverse variance weighted, LUAD = lung adenocarcinoma, OR = odds ratio, Treg = regulatory T cell.

We initially conducted a two-sample MR analysis of 412 GM and the risk of LUAD in order to evaluate the effect of numerous GMs on the risk of LUAD. The results of univariate MR analysis showed that high levels of PWY-5989 stearate biosynthesis II bacteria and plants (PWY-5989) decreased the risk of LUAD (IVW: *P* = .003, odds ratio [OR] = 0.640, 95% confidence interval [CI] = 0.476–0.861; Fig. 2). Meanwhile, the weighted median approaches yielded similar risk estimations (*P* = .019, OR = 0.647, 95% CI = 0.449–0.933). The *P*-values of the MR-Egger intercept and MR-PRESSO global test show no signs of directional pleiotropy (.362 and .348, respectively). Heterogeneity was not recognized, with a Cochran *Q* test derived *P*-value as .285 of IVW. The results of BWMR showed the similar risk estimates (*P* = .004, OR = 0.615, 95% CI = 0.438–0.862).

### 3.2. Effect of LUAD on PWY-5989

To explore the possibility of reverse causality, we performed reverse MR analyses. The data indicated a lack of reverse causal relationship between LUAD on PWY-5989 (IVW: *P* = .101, OR = 0.948, 95% CI = 0.890–1.010; MR-Egger: *P* = .713, OR = 0.959, 95% CI = 0.770–1.193; weighted median: *P* = .102, OR = 0.943, 95% CI = 0.880–1.011; Fig. 2).

### 3.3. Effect of PWY-5989 on immune cells

To explore the impact of PWY-5989 on immune cells, we performed a two-sample MR analysis of PWY-5989 and the risk of 731 immune cells. The results showed that high levels of PWY-5989 decreased the risk of CD25 on resting regulatory T cell (Treg; IVW: *P* = .010, OR = 0.720, 95% CI = 0.560–0.926; Fig. 2). Meanwhile, similar risk estimates were gained using the weighted median approaches (*P* = .028, OR = 0.696, 95% CI = 0.503–0.963). The *P*-values of the MR-Egger intercept and MR-PRESSO global test show no signs of directional pleiotropy (.349 and .285, respectively). Heterogeneity was not observed with a Cochran *Q* test derived *P*-value as .314 of IVW.

### 3.4. Effect of CD25 on resting Treg on LUAD

To investigate the effect of CD25 on resting Treg on LUAD, we conducted a two-sample MR analysis of CD25 on resting Treg and LUAD risk. Although other methods reflect no statistical significance, the IVW approach revealed increased CD25 on resting Treg was associated with a lower risk of LUAD (*P* = .002, OR = 0.886, 95% CI = 0.820–0.957; Fig. 2). No horizontal pleiotropy and heterogeneity were observed.

### 3.5. Mediation effect of PWY-5989 on LUAD

**Figure 3.**
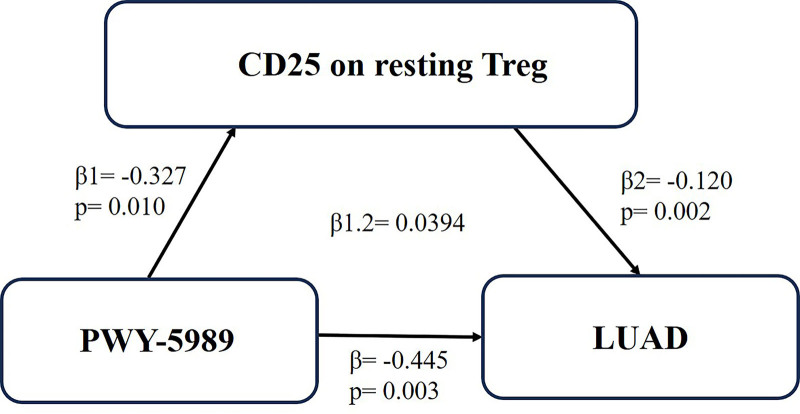
The CD25 on resting Treg mediated the causal effect of PWY-5989 on LUAD. LUAD = lung adenocarcinoma, Treg = regulatory T cell.

Finally, we conducted a mediation analysis to portray the mediation effect of CD25 on resting Treg between PWY-5989 and LUAD (Table 1). The mediation effect of CD25 on resting Treg from PWY-5989 to LUAD was 0.0394 (Fig. 3). In the presence of CD25 on resting Treg, PWY-5989 can promote the risk of LUAD by inhibiting the expression of CD25 on resting Treg.

**Table 1:** Mediation effect of PWY-5989 on LUAD via CD25 on resting Treg.

Total effect	Direct effect1	Direct effect2	Mediation effect
β	β1	β2	β1.2
−0.445	−0.327	−0.120	0.0394

Total effect (β): The causal role of PWY-5989 on LUAD. Direct effect 1 (β1): The causal role of PWY-5989 on CD25 on resting Treg. Direct effect 2 (β2): The causal role of CD25 on resting Treg on LUAD. Mediation effect (β1.2) = β (Direct effect 1) * β (Direct effect 2).

## 4. Discussion

An increasing body of studies have shown that the GM may be involved in the development and progression of various types of cancer, such as breast cancer,^[33]^ gastrointestinal cancer,^[34]^ and lung cancer.^[35]^ LUAD is the most common subtype of lung cancer. Due to technical limitations and lack of sufficient clinical data, the mechanisms by which bacterial metabolites drive LUAD remain unclear. Limited studies of the GM in LUAD patients have been performed. Sheng Wang et al found that compared to healthy individuals, the GM profile has significantly changed in patients, hinting that GM may be related to the progression of LUAD.^[36]^ “Gut-lung axis” is a novel idea, which suggests that the GM may influence lung homeostasis and susceptibility to lung diseases by regulating the immune system, metabolism, and endocrine, thus affecting the occurrence and development of lung cancer.^[6,37]^

Our study aimed to explore the causal effects between GM and LUAD. We performed two-sample MR analyses to investigate the causal effect of GM and LUAD based on existing GWAS and to demonstrate whether the causal relationship between them is mediated through immune cells. Firstly, we found the causality of PWY-5989 on LUAD was significant. Secondly, we found no reverse causality between PWY-5989 and LUAD through reverse MR analysis. To explore whether PWY-5989 regulate the immune system to affect NSCLC, we conducted a two-step MR study. Thirdly, we analyzed the causal effects of PWY-5989 on 731 immune cells. The results indicated that high levels of PWY-5989 decreased the risk of CD25 on resting Treg. Then, we performed a two-sample MR analysis of CD25 on resting Treg and the risk of LUAD to explore the impact of CD25 on resting Treg on LUAD. The results revealed increased CD25 on resting Treg was associated with a lower risk of LUAD. Finally, we calculated the results of indirect effects, direct effects using mediation analyses. The data showed the mediation effect of CD25 on resting Treg was 0.0394, hinting that CD25 on resting Treg was a crucial mediator in the relationship between PWY-5989 and LUAD risk.

A large number of studies have indicated that abnormal lipid metabolism may be closely related to tumor development.^[38–41]^ But which fatty acids metabolic processes play a role in tumorigenesis remains unclear. Stearate (C18:0) is a dietary long chain saturated fatty acid. Previous study indicated that stearate inhibits tumor development in rats.^[42]^ Some studies have shown that stearate can inhibit breast cancer progression.^[43–45]^ However, until now, no studies have shown a relationship between the stearate biosynthesis pathway and LUAD. CD25 is the α-chain of the heterotrimer IL-2 receptor and highly expressed in Tregs.^[46]^ In the tumor microenvironment, infiltration of Tregs can cause an imbalance of effector T cells (Teffs) and Tregs, which are associated with the progression of cancer.^[47]^ GM can play an important role in immune response.^[48]^ Herein, we demonstrated that CD25 on resting Treg may be a critical moderator between PWY-5989 and LUAD.

In the study, we explored the causal role of GM and LUAD by MR analysis, as well as the mediating role of immune cells in the association between GM and LUAD. Our study has several strengths. First, through the MR study, we can simulate randomized controlled trials in an observational setting. Second, our findings may guide early screening and prevention policies for LUAD. Third, the identified causal pathway (PWY-5989/CD25 on resting Treg/LUAD) highlights 2 potential nodes for risk stratification. Microbiome-derived metabolites related to stearate biosynthesis or peripheral CD25 expression on Tregs could be explored as novel, noninvasive biomarkers for LUAD susceptibility. Fourth, from a therapeutic perspective, our results lend causal support to the “gut-lung axis” hypothesis. This suggests that interventions aimed at modulating the gut microbiome or fine-tuning Treg activity might represent promising strategies for LUAD prevention or as adjuvants to existing therapies. However, it is important to note that there were still several limitations. First, despite employing multiple sensitivity analyses, the potential for residual horizontal pleiotropy cannot be entirely ruled out, which is an inherent limitation of MR studies. Second, our analysis utilized microbiome data at the genus and pathway levels; future investigations incorporating strain-level data or specific microbial metabolites could provide finer mechanistic insights. Third, the observed mediation effect, while significant, was modest in magnitude, suggesting that other immune or nonimmune mediators may also contribute to the pathway from GM to LUAD, warranting further exploration. Fourth, our MR analyses were conducted using summary-level GWAS data predominantly from individuals of European ancestry. While this minimizes population stratification bias, it may limit the generalizability of our findings to other ethnic groups. Future studies utilizing genetic and epidemiological data from diverse populations are warranted to validate and extend the causal relationships identified herein.

## 5. Conclusion

Our study illustrated the causal relationships between GM, immune cells, and LUAD. Specifically, PWY-5989 could increase the risk of LUAD, which was, to a large proportion, mediated by CD25 on resting Treg.

## Acknowledgments

We are grateful to the consortium that provided all the public GWAS data.

## Author contributions

**Conceptualization:** Lishi Yang, Bin Liang, Fuhua Sun.

**Data curation:** Lishi Yang, Lei Li.

**Formal analysis:** Jihua Yu.

**Funding acquisition:** Lishi Yang, Fuhua Sun.

**Investigation:** Bin Liang.

**Methodology:** Lishi Yang, Lei Li, Fuhua Sun.

**Software:** Lishi Yang, Lei Li.

**Supervision:** Bin Liang, Jihua Yu, Jianxiong Wang.

**Visualization:** Jianxiong Wang, Fuhua Sun.

**Writing – original draft:** Lei Li.

**Writing – review & editing:** Fuhua Sun.
